# Supplementary figures and images for: Proteasome activator 28γ (PA28γ) allosterically activates trypsin-like proteolysis by binding to the α-ring of the 20S proteasome
Source: J Biol Chem. 2022 Jun 14;298(8):102140. doi: 10.1016/j.jbc.2022.102140 (PMC9287138; doi:10.1016/j.jbc.2022.102140)

Supplemental Figure 1:

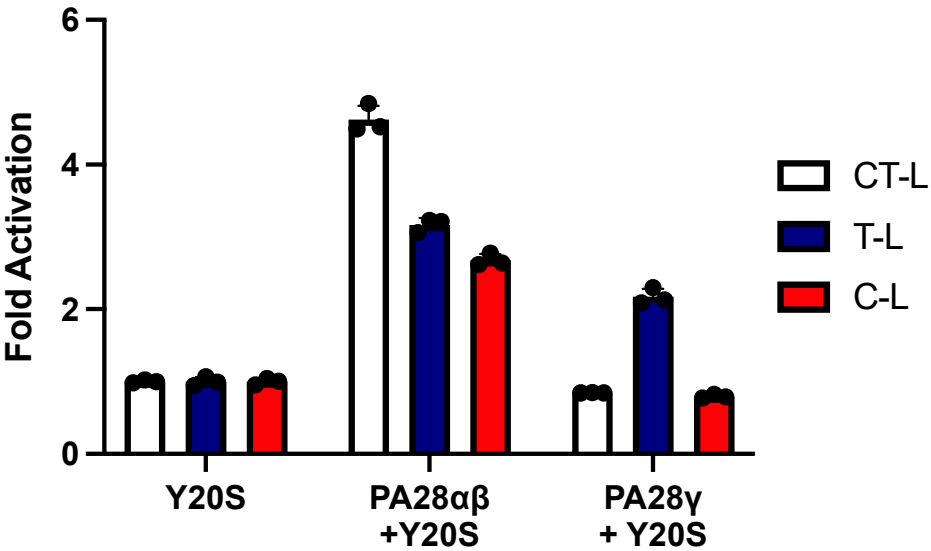

Supplement: Supplementary Figure 1 — WT Y20S proteasome activity assay. Proteasome activity assay with PA28αβ and PA28γ reveals a similar pattern of activation between Y20S and H20S (Fig 1C). Purified Y20S (1nM) was tested for all three proteolytic sites (RFU/min) in the presence of recombinant PA28αβ (50nM), and PA28γ (62.5nM). Results are the mean of at least three independent experiments performed in triplicate (error bars represent SD) normalized to the Y20S control. PA28γ, proteasome activator 28γ; H20S, human 20S proteasome. [file mmc1.pdf]

Supplemental Figure 2:

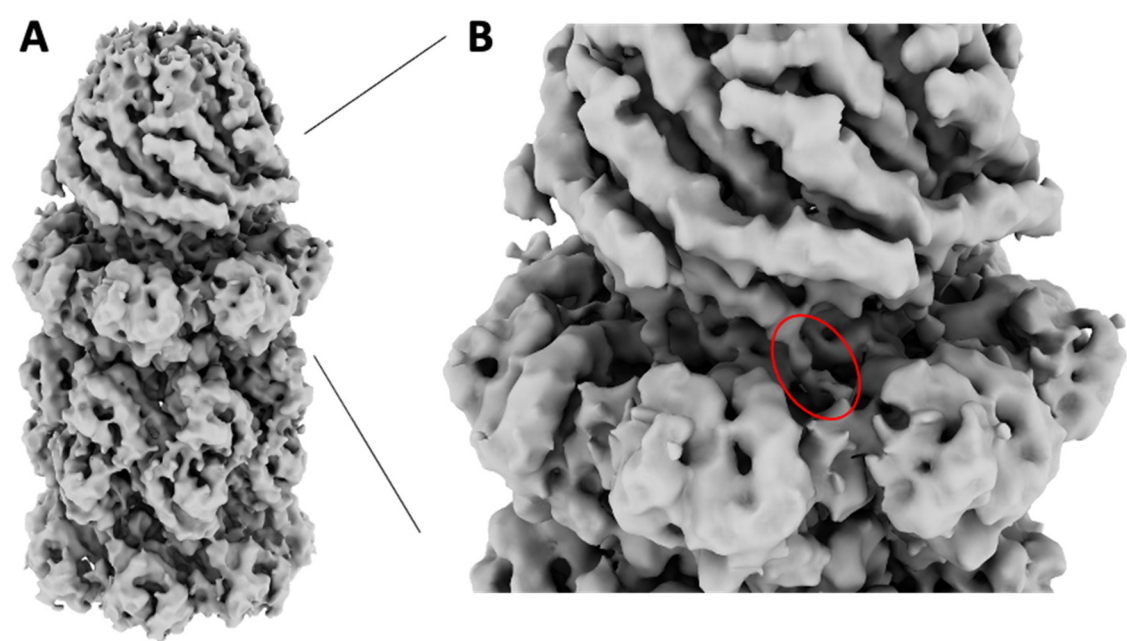

Supplement: Supplementary Figure 2 — Cryo-EM map and C-terminal docking site. PA28γ-20S complex shows C termini are docked into the intersubunit pockets of the 20S. A, complete side view; B, zoomed view of “A” shown by lines. Red circle highlights one of PA28γ’s 7 C termini docking into one 20S intersubunit pocket. PA28γ, proteasome activator 28γ. [file mmc2.pdf]

# Supplemental Figure 3:

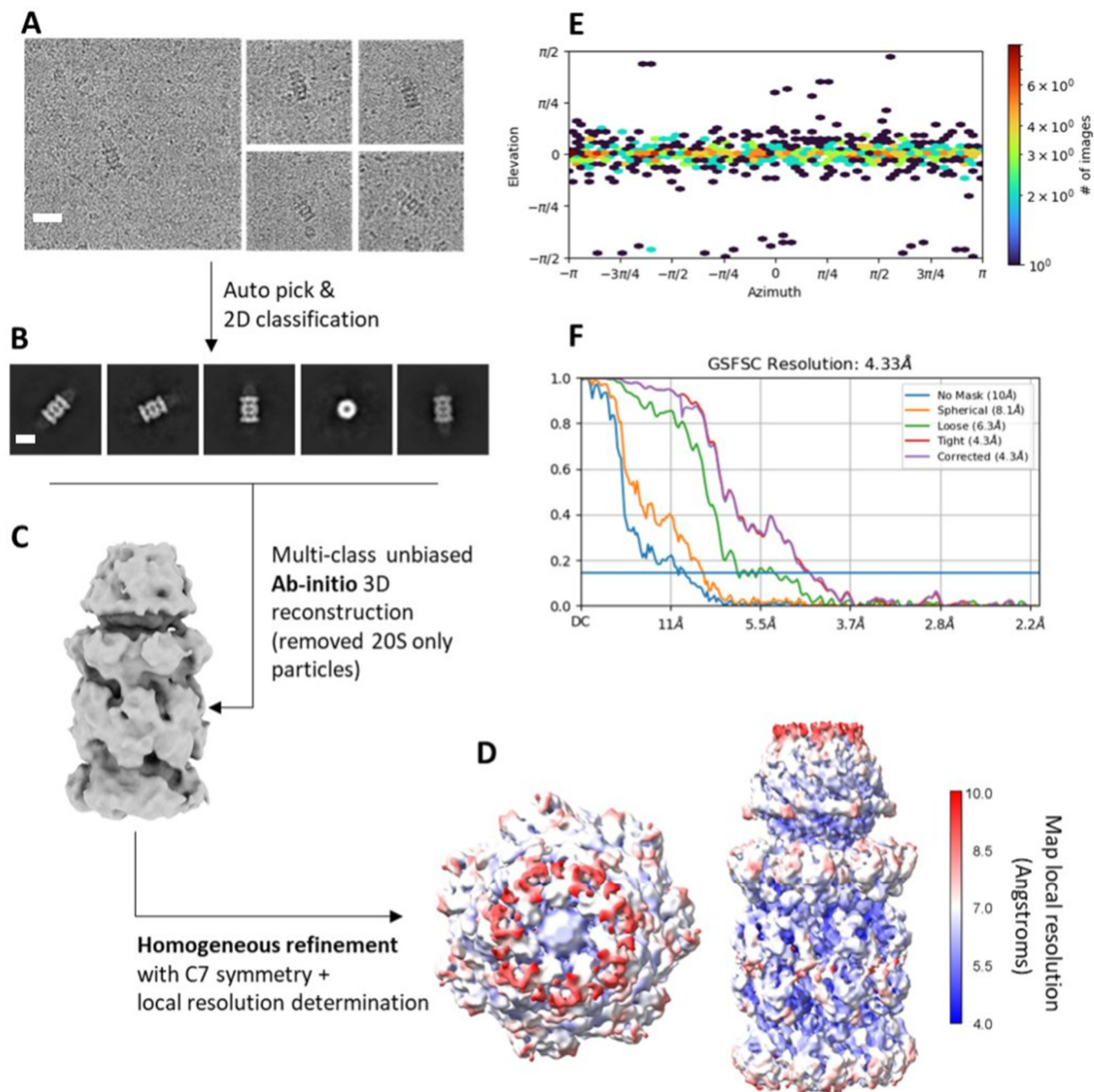

Supplement: Supplementary Figure 3 — Cryo-EM workflow and validation.A, raw motion corrected images of sparsely located PA28γ-20S complexes. Scale bar represents 20nm. B, several rounds of 2D classification were done to remove junk particles and the final 2D class averages are shown with mostly side views of single and double caped PA28γ-20S complexes and a single top view. Scale bar represents 15nm. C, unbiased ab-initio 3D reconstruction with five classes was run to further separate 20S particles from PA28γ-20S complexes (no symmetry was applied at this step). One class contained a clear PA28γ-20S complex (shown) that was derived from 876 particles. D, particles from the selected ab-initio model were refined with a homogeneous refinement job (with C7 symmetry). The resulting 4.4Å map was colored with outputs from the local resolution job (cryoSPARC) in Chimera to show resolution variability of 3D reconstructed sharpened map. Map resolution colored according to the scale that is shown. E, view of particle angle distribution showing the vast majority of particles that were used for 3D reconstruction were side view. F, gold standard FSC-0.143 graph showing corrected 4.3Å resolution. PA28γ, proteasome activator 28γ. [file mmc3.pdf]
