# Supplementary material for: Proteasome activator 28γ (PA28γ) allosterically activates trypsin-like proteolysis by binding to the α-ring of the 20S proteasome
Source: J Biol Chem. 2022 Jun 14;298(8):102140. doi: 10.1016/j.jbc.2022.102140 (PMC9287138; doi:10.1016/j.jbc.2022.102140)
Supplement: val-report-full-annotate [file mmc4.pdf]

Full wwPDB EM Validation Report (i)

Mar 7, 2022 – 11:38 AM EST

EMDB ID	:	EMD-26379
Title	:	Structure of PA28gamma bound to the human 20S proteasome with C7 symmetry.
Deposited on	:	2022-03-05
Resolution	:	4.40 Å(reported)

This wwPDB validation report is for manuscript review

For Manuscript Review

This is a Full wwPDB EM Validation Report.

This report is produced by the wwPDB biocuration pipeline after annotation of the structure.

We welcome your comments at *validation@mail.wwpdb.org*
A user guide is available at
https://www.wwpdb.org/validation/2017/EMMapValidationReportHelp
with specific help available everywhere you see the (i) symbol.

The following versions of software and data (see references (i)) were used in the production of this report:

EMDB validation analysis	:	0.0.0.dev97
Validation Pipeline (wwPDB-VP)	:	2.27

1 Experimental information (i)

Property	Value	Source
EM reconstruction method	SINGLE PARTICLE	Depositor
Imposed symmetry	POINT, C7	Depositor
Number of particles used	876	Depositor
Resolution determination method	FSC 0.143 CUT-OFF	Depositor
CTF correction method	PHASE FLIPPING AND AMPLITUDE CORRECTION	Depositor
Microscope	FEI TITAN KRIOS	Depositor
Voltage (kV)	300	Depositor
Electron dose $(e^-/\text{Å}^2)$	50	Depositor
Minimum defocus (nm)	Not provided	
Maximum defocus (nm)	Not provided	
Magnification	Not provided	
Image detector	FEI FALCON III (4k x 4k)	Depositor
Maximum map value	1.450	Depositor
Minimum map value	-0.661	Depositor
Average map value	0.002	Depositor
Map value standard deviation	0.063	Depositor
Recommended contour level	0.64	Depositor
Map size (Å)	552.96, 552.96, 552.96	wwPDB
Map dimensions	512, 512, 512	wwPDB
Map angles (°)	90.0, 90.0, 90.0	wwPDB
Pixel spacing (Å)	1.08, 1.08, 1.08	Depositor

For Manuscript Review

2 Map visualisation (i)

This section contains visualisations of the EMDB entry EMD-26379. These allow visual inspection of the internal detail of the map and identification of artifacts.

No raw map or half-maps were deposited for this entry and therefore no images, graphs, etc. pertaining to the raw map can be shown.

2.1 Orthogonal projections (i)

2.1.1 Primary map

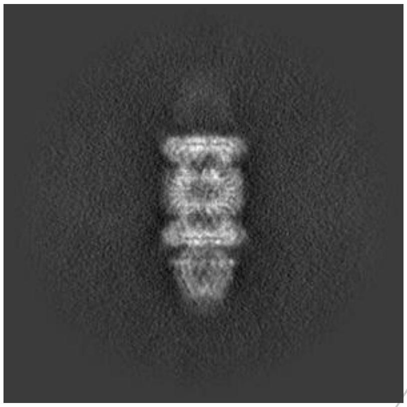

X

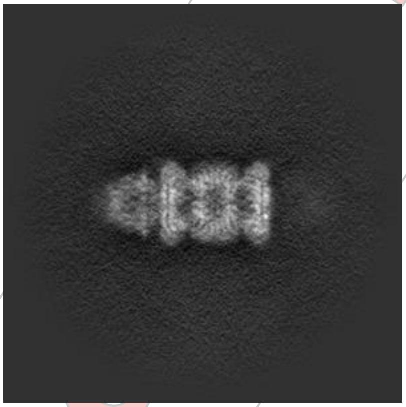

Y

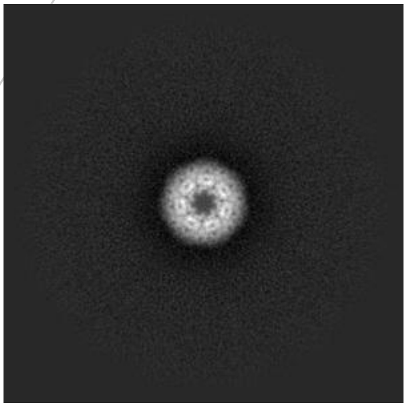

Z

The images above show the map projected in three orthogonal directions.

2.2 Central slices (i)

2.2.1 Primary map

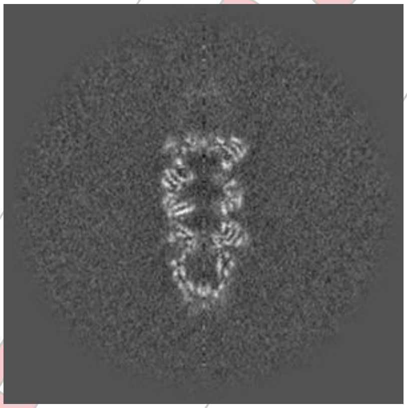

X Index: 256

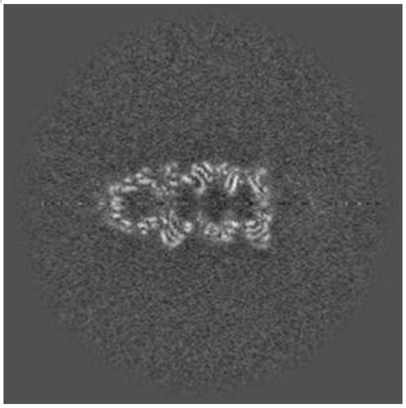

Y Index: 256

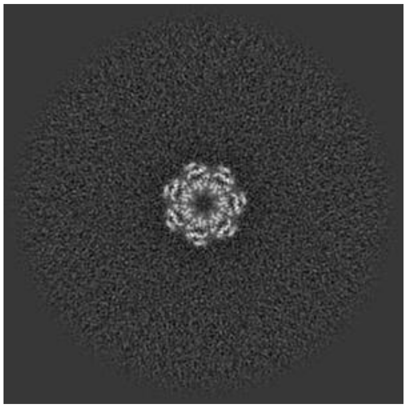

Z Index: 256

For Manuscript Review

The images above show central slices of the map in three orthogonal directions.

2.3 Largest variance slices (i)

2.3.1 Primary map

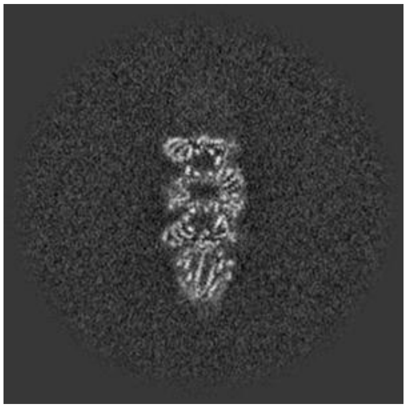

X Index: 238

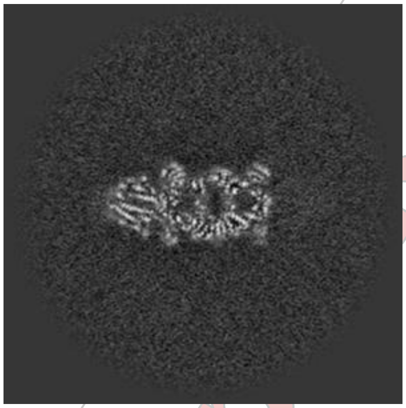

Y Index: 237

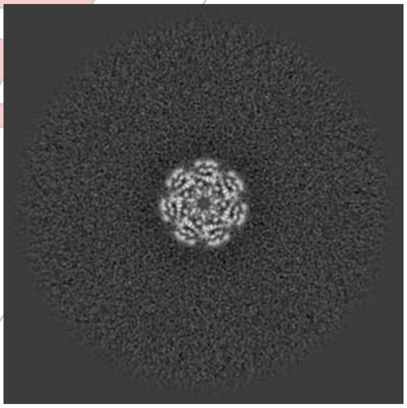

Z Index: 217

The images above show the largest variance slices of the map in three orthogonal directions.

2.4 Orthogonal surface views (i)

2.4.1 Primary map

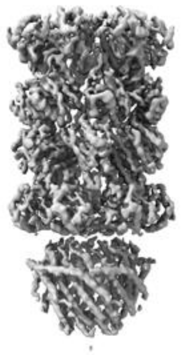

X

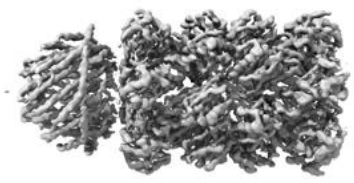

Y

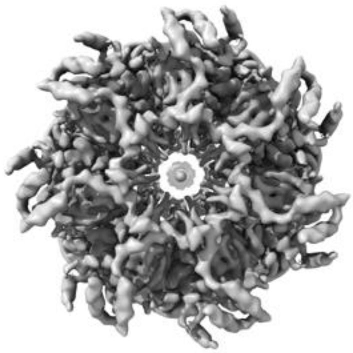

Z

The images above show the 3D surface view of the map at the recommended contour level 0.64. These images, in conjunction with the slice images, may facilitate assessment of whether an appropriate contour level has been provided.

For Manuscript Review

2.5 Mask visualisation ⓘ

This section was not generated. No masks/segmentation were deposited.

For Manuscript Review

3 Map analysis (i)

This section contains the results of statistical analysis of the map.

3.1 Map-value distribution (i)

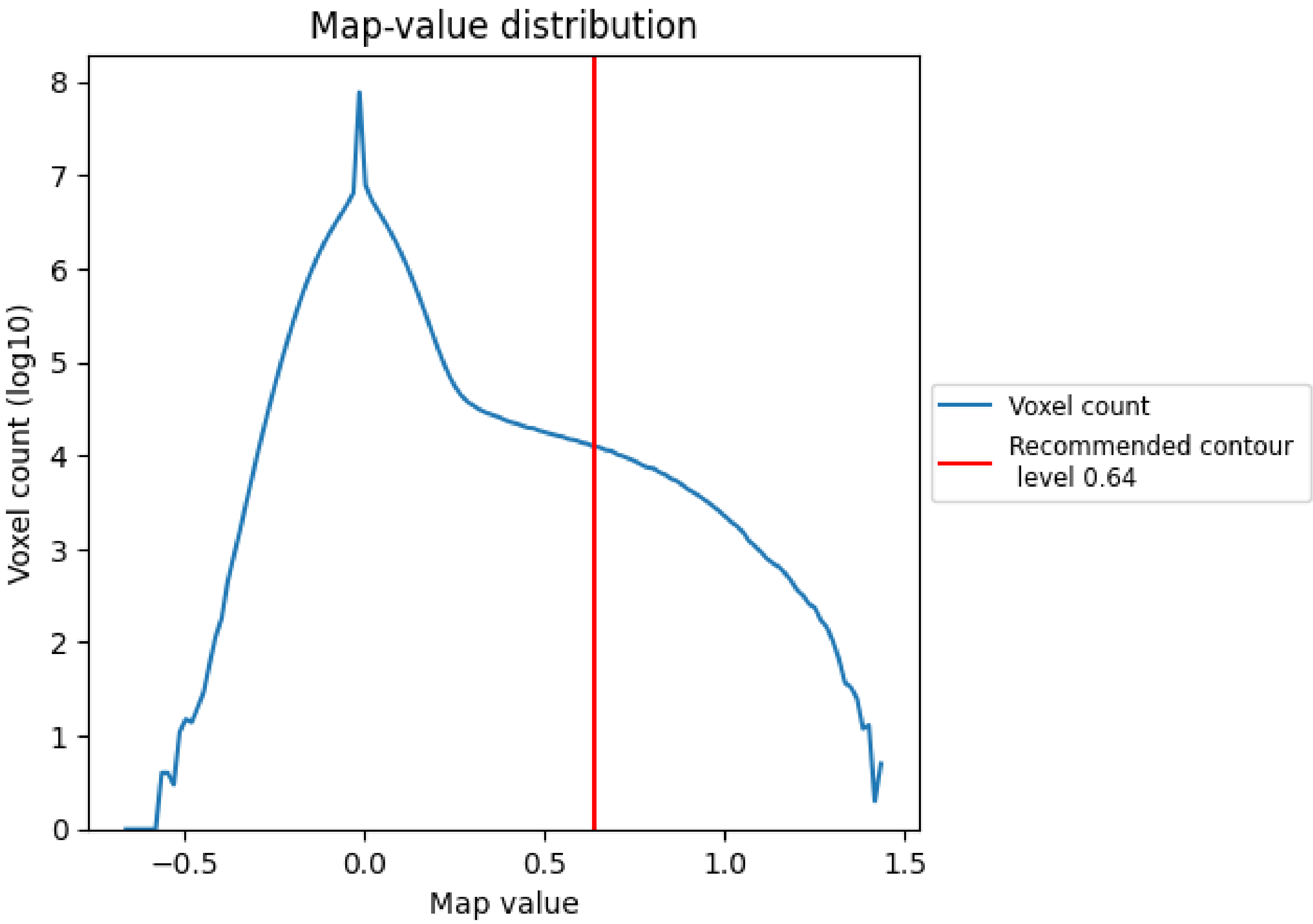

The map-value distribution is plotted in 128 intervals along the x-axis. The y-axis is logarithmic. A spike in this graph at zero usually indicates that the volume has been masked.

For Manuscript Review

3.2 Volume estimate (i)

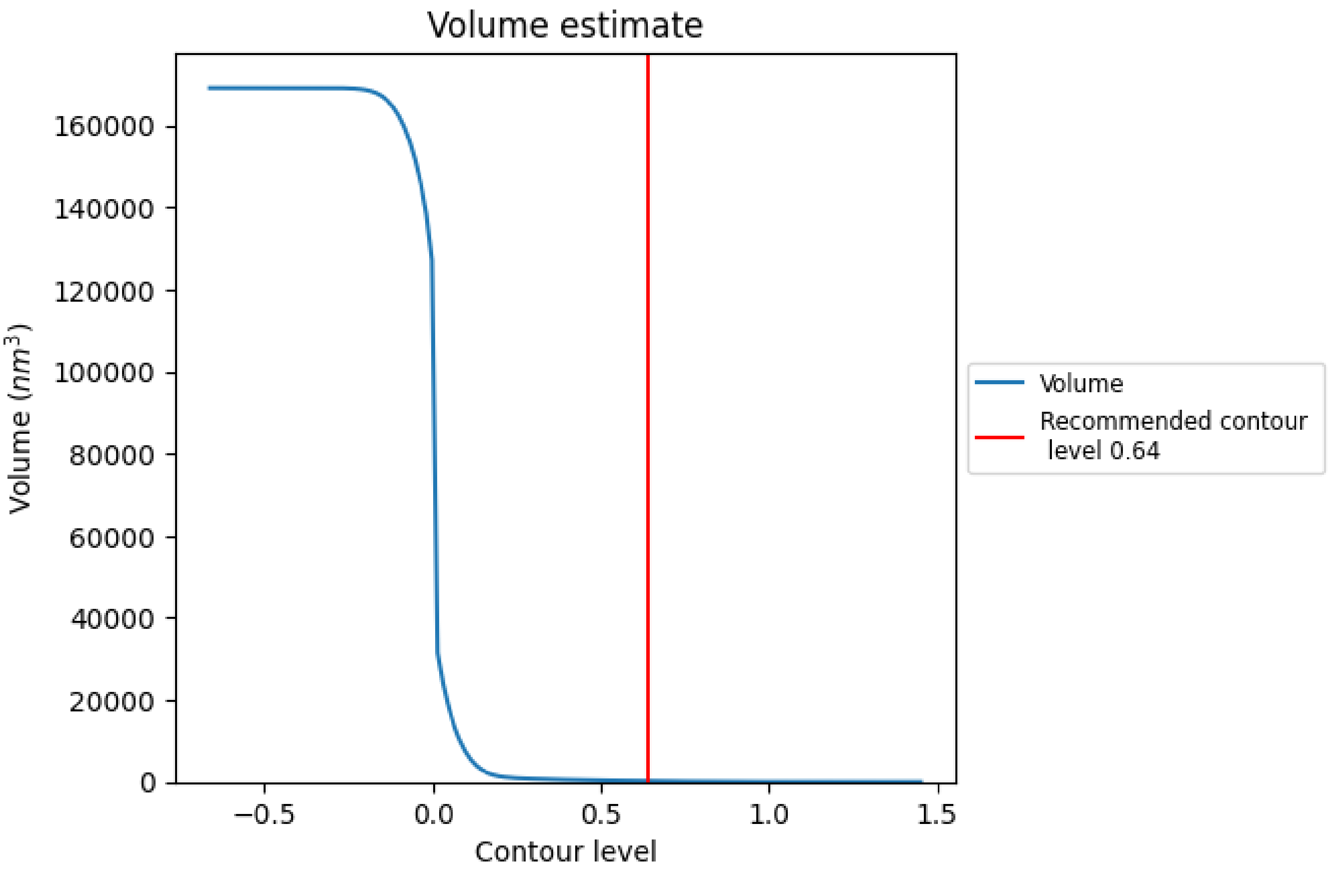

The volume at the recommended contour level is 213 nm^3; this corresponds to an approximate mass of 192 kDa.

The volume estimate graph shows how the enclosed volume varies with the contour level. The recommended contour level is shown as a vertical line and the intersection between the line and the curve gives the volume of the enclosed surface at the given level.

For Manuscript Review

3.3 Rotationally averaged power spectrum (i)

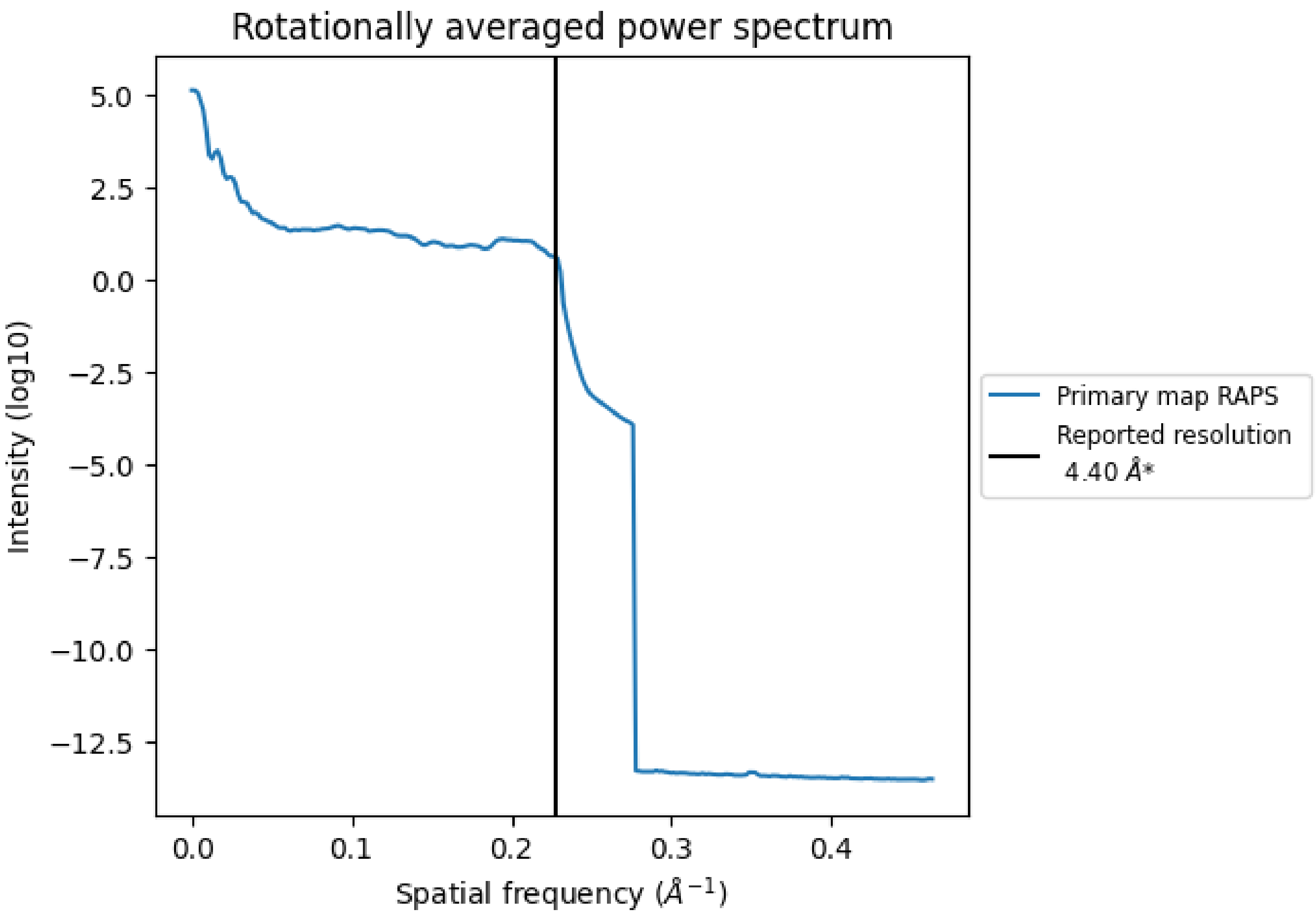

*Reported resolution corresponds to spatial frequency of 0.227 Å^{-1}

For Manuscript Review

4 Fourier-Shell correlation (i)

Fourier-Shell Correlation (FSC) is the most commonly used method to estimate the resolution of single-particle and subtomogram-averaged maps. The shape of the curve depends on the imposed symmetry, mask and whether or not the two 3D reconstructions used were processed from a common reference. The reported resolution is shown as a black line. A curve is displayed for the half-bit criterion in addition to lines showing the 0.143 gold standard cut-off and 0.5 cut-off.

4.1 FSC (i)

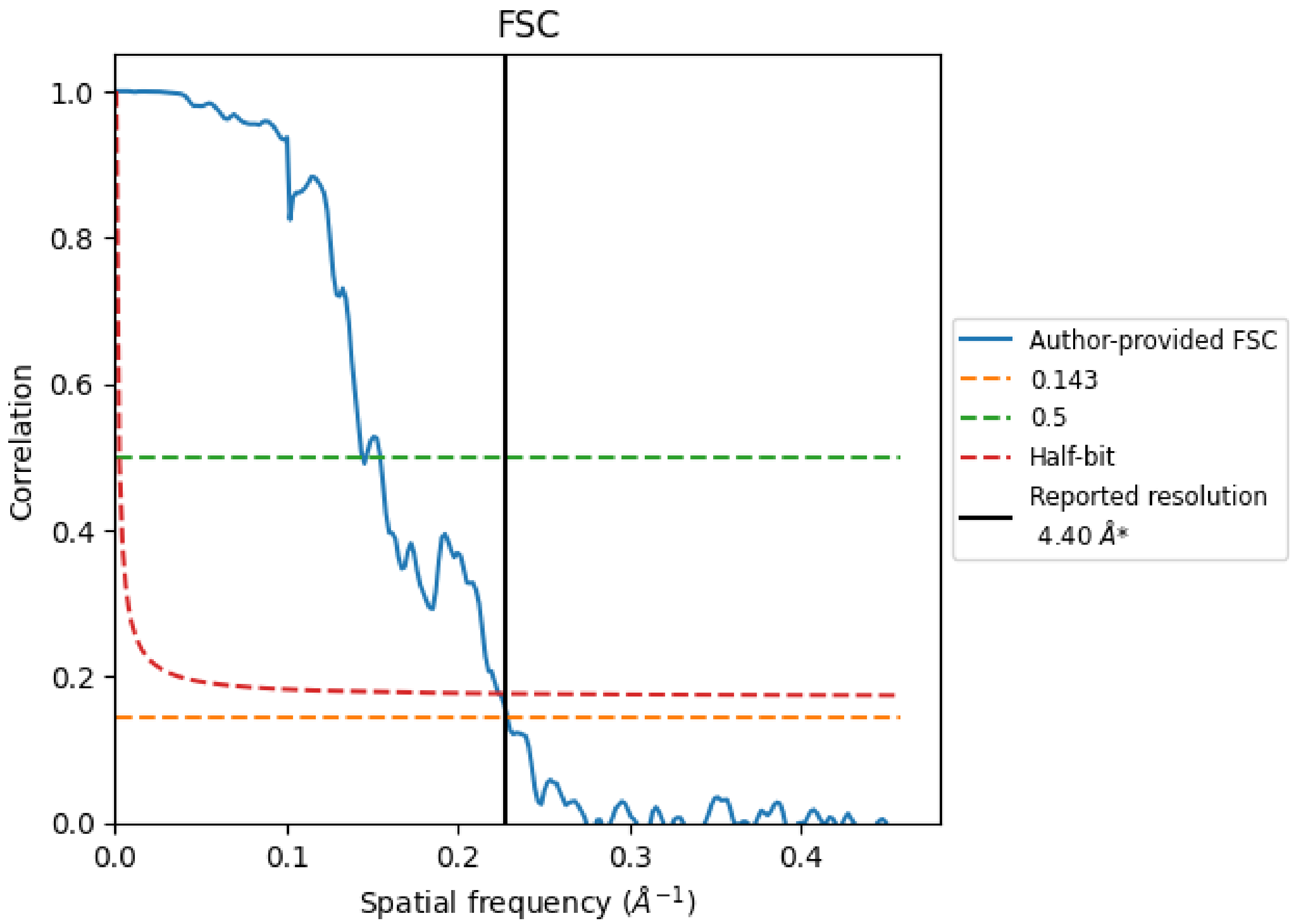

*Reported resolution corresponds to spatial frequency of 0.227 Å^{-1}

For Manuscript Review

4.2 Resolution estimates (i)

Resolution estimate (Å)	Estimation criterion (FSC cut-off)		
	0.143	0.5	Half-bit
Reported by author	4.40	-	-
Author-provided FSC curve	4.37	6.92	4.46
Unmasked-calculated*	-	-	-

*Resolution estimate based on FSC curve calculated by comparison of deposited half-maps.

For Manuscript Review
